# Therapeutic efficacy of artemether-lumefantrine for the treatment of uncomplicated *Plasmodium falciparum* malaria in four malaria endemic states of India

**DOI:** 10.1186/s12936-021-03762-7

**Published:** 2021-05-21

**Authors:** Sri Krishna, Sweta Mishra, Prakash Tiwari, Anup K. Vishwakarma, Sushrikanta Khandai, Suyesh Shrivastava, Anil K. Verma, Shashikant Tiwari, Hari Barman, Surendra Jhariya, Pradeep Tiwari, Anup S. Tidgam, Brij M. Varun, Sunil Singh, Naresh Yerane, Chintaman R. Tembhurne, Prem L. Mandavi, Shyam S. Tekam, Manas Malik, Kali P. Behera, Himanshu Jayswar, Khemraj Sonwani, Mukund S. Diggikar, Madan M. Pradhan, Sher S. Khasotiya, Avdhesh Kumar, Neeraj Dhingra, Maria Dorina G. Bustos, Eva-Maria Christophel, Pascal Ringwald, Roop Kumari, Man M. Shukla, Neeru Singh, Aparup Das, Praveen K. Bharti

**Affiliations:** 1 ICMR-National Institute of Research in Tribal Health, Jabalpur, Madhya Pradesh India; 2 Medical Officer, Balaghat, Madhya Pradesh India; 3 District Malaria Office, Balaghat, Madhya Pradesh India; 4 Community Health Centre Damoh, Balaghat, Madhya Pradesh India; 5 Community Health Centre Darekasa, Gondia, Maharashtra India; 6 District Malaria Office, Gondia, Maharashtra India; 7 Community Health Centre, District Bastar, Darbha, Chhattisgarh India; 8 District Malaria Office, District Bastar, Jagdalpur, Chhattisgarh India; 9 Community Health Centre Bandhgram, District Koraput, Dasmantpur, Odisha India; 10 District Malaria Office, District Koraput, Dasmantpur, Odisha India; 11 Directorate of Health Services, Satpura Bhawan, Bhopal, Madhya Pradesh India; 12 Directorate of Health Services, Indravati Bhawan, Raipur, Chhattisgarh India; 13 Arogya Bhavan, Yerawada, Pune, Maharashtra India; 14 State NVBDCP, Public Health Directorate, Bhubaneswar, Odisha India; 15 National Vector Borne Disease Control Programme (NVBDCP), New Delhi, India; 16 Country Office for Thailand, World Health Organization, Bangkok, Thailand; 17 World Health Organization, Regional Office for South-East Asia, New Delhi, India; 18 Global Malaria Programme, World Health Organization, Geneva, Switzerland; 19 World Health Organization, Country Office for India, New Delhi, India

**Keywords:** Therapeutic efficacy, Artemether-lumefantrine, *Plasmodium falciparum*, Malaria

## Abstract

**Background:**

Malaria is a major public health problem in India and accounts for about 88% of malaria burden in South-East Asia. India alone accounted for 2% of total malaria cases globally. Anti-malarial drug resistance is one of the major problems for malaria control and elimination programme. Artemether-lumefantrine (AL) is the first-line treatment of uncomplicated *Plasmodium falciparum* in north eastern states of India since 2013 after confirming the resistance against sulfadoxine-pyrimethamine. In the present study, therapeutic efficacy of artemether-lumefantrine and *k13* polymorphism was assessed in uncomplicated *P. falciparum* malaria.

**Methods:**

This study was conducted at four community health centres located in Koraput district of Odisha, Bastar district of Chhattisgarh, Balaghat district of Madhya Pradesh and Gondia district of Maharashtra state. Patients with uncomplicated *P. falciparum* malaria were administered with fixed dose combination (6 doses) of artemether-lumefantrine for 3days and clinical and parasitological response was recorded up to 28days as per World Health Organization protocol. Nucleotide sequencing of *msp1* and *msp2* gene was performed to differentiate between recrudescence and reinfection. Amplification and sequencing of *k13* propeller gene region covering codon 450680 was also carried out to identify the polymorphism.

**Results:**

A total 376 malaria patients who fulfilled the enrolment criteria as well as consented for the study were enrolled. Total 356 patients were followed up successfully up to 28days. Overall, the adequate clinical and parasitological response was 98.9% and 99.4% with and without PCR correction respectively. No case of early treatment failure was observed. However, four cases (1.1%) of late parasitological failure were found from the Bastar district of Chhattisgarh. Genotyping of *msp1* and *msp2* confirmed 2 cases each of recrudescence and reinfection, respectively. Mutation analysis of k13 propeller gene showed one non-synonymous mutation Q613H in one isolate from Bastar.

**Conclusions:**

The study results showed that artemether-lumefantrine is highly effective in the treatment of uncomplicated *P. falciparum* malaria among all age groups. No functional mutation in *k13* was found in the study area. The data from this study will be helpful in implementation of artemether-lumefantrine in case of treatment failure by artesunate plus sulfadoxine-pyrimethamine.

## Background

Malaria is a major public health problem in India and accounts for about 88% of malaria burden in South-East Asia. India alone accounted for 2% of total malaria cases globally [1]. In India, a total of 3,38,494 malaria cases was reported in year 2019 and about 46.4% of malaria cases were caused by *Plasmodium falciparum* monoinfections [2]. Anti-malarial drug resistance is one of the major problems for malaria control and elimination programme [3].

First information of chloroquine resistant *P. falciparum* was reported from Assam in 1973 within India [4], and resistance gradually spread to other states covering almost entire country [5]. Emergence of sulfadoxine-pyrimethamine (SP) resistance was also reported in Karbi-Anglong, district of Assam in 1979 [6]. SP resistance was again reported in 1992 from Changlang district of Arunachal Pradesh, a state of north-east India [7]. Later artemisinin-based combination therapy (ACT), using artesunate plus sulfadoxine-pyrimethamine (AS+SP), was introduced as the second-line drug in 2005, in case of chloroquine treatment failures. In 2007, AS+SP was selected as the first-line treatment in areas with identified resistance [8]. In 2010, this treatment became the first-line treatment throughout India [9]. Later, due to reports of resistance to partner drug SP in North Eastern States, Co-formulated tablet of artemether-lumefantrine (AL) was introduced by programme for the treatment of *P. falciparum* cases [10].

Fixed dose combination of artemether (20mg) and lumefantrine (120mg) is commercially available. Artemether has a half-life of 13h, as it absorbs and metabolizes quickly leading to fast clearance of parasite from blood. Due to longer half-life of lumefantrine (36days), it further eliminates the residual parasite from the malaria patient and helps to prevent the recrudescence [11]. Therefore, combination of artemether and lumefantrine reduces the chance of parasite to develop resistance.

Mutation in *k13* propeller gene has found to be associated with artemisinin resistance in Southeast Asia [12]. Many mutations Y493H, R539T, I543T, and C580Y are known which are responsible for in vivoandin vitroartemisinin resistance, while P553L is known for delayed parasite clearance in Southeast Asia [13]. India is a major contributor to the malaria load in Southeast Asia. Therefore, it is also important to keep track of emerging alleles of *k13* in India.

Periodic monitoring of therapeutic efficacy is recommended by World Health Organization (WHO) to control the malaria and avoid the spread of resistant parasite in the population. Therefore, therapeutic efficacy of AL for the treatment of uncomplicated *P. falciparum* malaria was assessed in four states, Madhya Pradesh, Chhattisgarh, Maharashtra and Odisha and mutation in *k13* propeller gene was analysed to confirm the artemisinin resistance.

## Methods

### Study site details

**Fig. 1:**
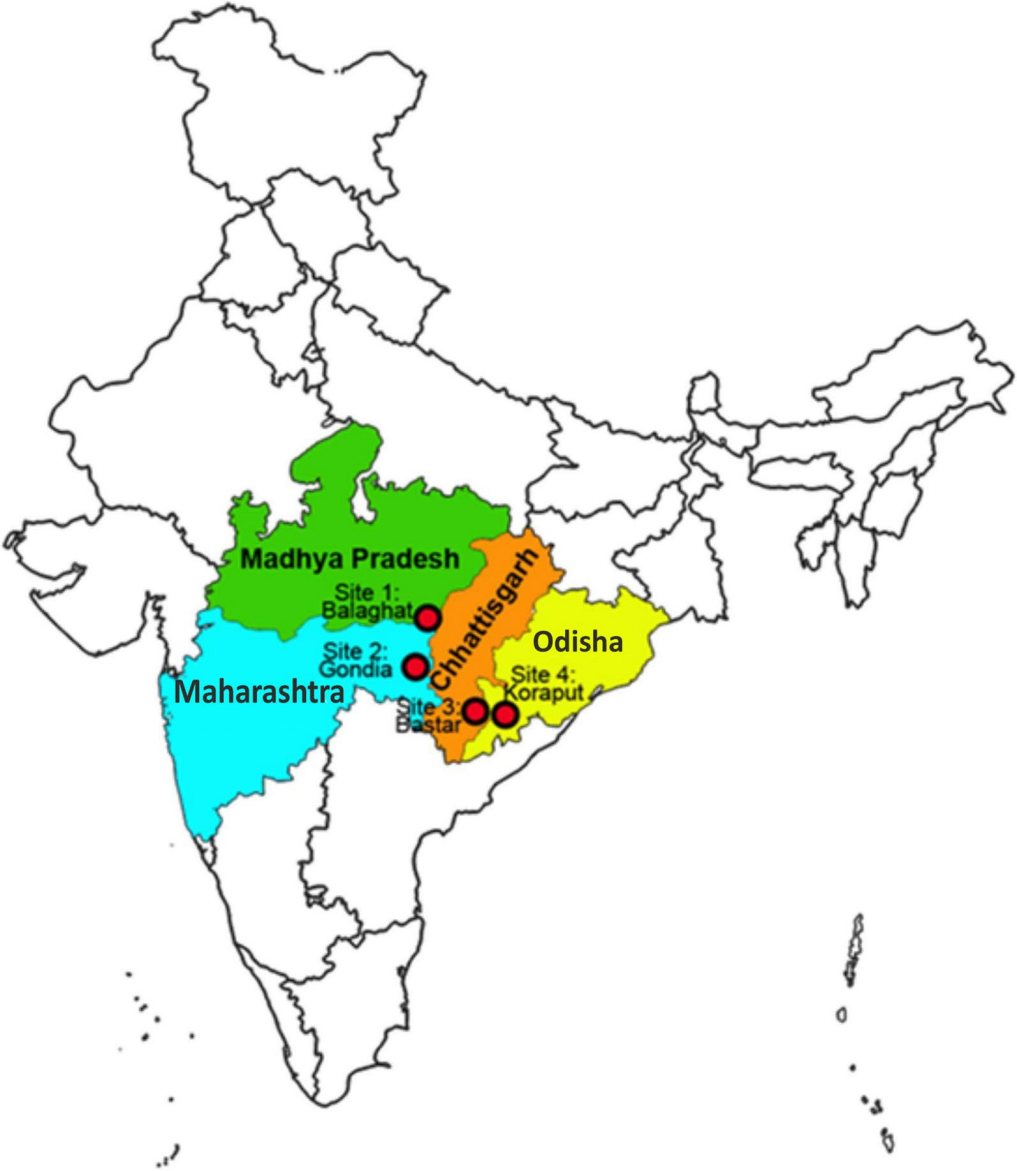
Map showing the study sites i.e. district Koraput (Odisha), district Bastar (Chhattisgarh), district Balaghat (Madhya Pradesh) and district Gondia (Maharashtra)

This study was a one-arm prospective study conducted at four sites during June 2017 to Dec 2017. These four community health centres (CHCs) located in Koraput district of Odisha, Baster district of Chhattisgarh, Balaghat district of Madhya Pradesh and Gondia district of Maharashtra state (Fig.1). Selection of these sites was based on malaria endemicity and geographic profile. These sites are tribal dominating located in remote forest and malaria is a major health problem in these areas. Both *P. falciparum* and *Plasmodium vivax* were reported from these sites. The selected CHCs were having sufficient facility for management of uncomplicated malaria. All these sites were at distance of 40 to 70km from their district headquarter for the management of severe malaria cases at district hospitals.

### Screening of malaria patients

Febrile patients aged between 6months and 60years of age attending the CHC hospitals were screened for malaria parasite by microscopy and patients with uncomplicated *P. falciparum* infection were asked to participate in the study. The unmarried female (1218years) were excluded from the study as the request for pregnancy test and initiation of contraceptive was not acceptable in local areas.

### Inclusion criteria

Symptomatic patients aged 6months (>5kg body weight) and 60years of age with uncomplicated malaria due to monoinfection of *P. falciparum* (parasitaemia of 1000 to 100,000/L asexual forms microscopically), fever or history of fever within 24 previous hours, ability to swallow oral medication and willing to comply with the study protocol for the duration of the study were included [14].

### Exclusion criteria

Patients with general danger signs or signs of severe falciparum malaria; severe malnutrition; mixed or monoinfections other than *P. falciparum* species as detected by microscopy; febrile conditions caused by a disease other than malaria or another known underlying chronic or severe disease; female patients with positive pregnancy test or breastfeeding, who were unable to drink, had severe vomiting, presence of lethargy or decreased consciousness, inability to sit or stand, were all excluded [14].

### Sample size

As the treatment failure rate to (artemether-lumefantrine) in the area is unknown, 5% was assumed. At a confidence level of 95% and a precision around the estimate of 5%, a minimum of 73 patients must be included. With a 20% increase to allow loss to follow-up and withdrawals during the 28day follow-up period, 88 patients were required in the study per site.

### Enrolment and sample collection

The patient screening and enrolment started from June 2017 till December 2017. Patients presenting with fever or history of fever and symptoms of malaria were screened for malaria parasites by microscopy. Patients positive for *P. falciparum* malaria and fulfilling the enrolment criteria were enrolled in the study. The demographic information (age, gender, body temperature) were recorded. Two to three drops of finger prick blood was also blotted on to 3 MM filter paper (Whatman International Ltd., Maidstone, UK) at the time of enrolment and during the follow-up for molecular study.

### Anti-malarial treatment with artemether-lumefantrine

Enrolled patients were clinically examined by on duty medical officer and artemether-lumefantrine tablets were administered orally according to body weight, twice a day over 3days (Make-Ipca, Batch No.-DYI576027, Expiry date-01/2018) as per Indian National Malaria Drug Policy 2013 [15]. Female patients were asked for pregnancy test as the drug is contraindicated in the first trimester of pregnancy. Clinical and parasitological parameters were monitored over a 28-day follow-up period to evaluate drug efficacy. The day patient was enrolled and administered with first dose of artemether-lumefantrine was mentioned as 0day. Patients were observed for a minimum 30min after intake of drug to ensure that there was no vomiting after treatment. After three days of completed treatment (0, 1, 2day), clinical and microscopic follow up examination of patients on 3, 7, 14, 21 and 28day was also performed and outcomes were recorded into the following categories according to the WHO protocol [14].

### Study definition

#### Early treatment failure (ETF)

Patient develops danger signs or severe malaria on Day 1, Day 2 or Day 3 and there are parasites in the blood, or the number of parasites in the blood on Day 2 is greater than that on the day of enrolment (Day 0), or axillary temperature on Day 3 is37.5C and there are parasites in the blood on Day 3, and Day 3 parasitaemia25% of those counted at the day of enrolment (Day 0).

#### Late clinical failure (LCF)

- Danger signs or severe malaria in the presence of parasitaemia on any day between day 4 and day 28 in patients who did not previously meet any of the criteria of early treatment failure; and
- Presence of parasitaemia on any day between day 4 and day 28 with axillary temperature37.5C in patients who did not previously meet any of the criteria of early treatment failure.

#### Late parasitological failure (LPF)

Presence of parasitaemia on any day between day 7 and day 28 with axillary temperature<37.5C in patients who did not previously meet any of the criteria of early treatment failure or late clinical failure.

#### Adequate clinical and parasitological response (ACPR)

Absence of parasitaemia on day 28, irrespective of axillary temperature, in patients who did not previously meet any of the criteria of early treatment failure, late clinical failure or late parasitological failure.

### Microscopic examination of blood

Counting of parasite was done on Giemsa-stained thick blood films until 200 white blood cells (WBCs) counted by light microscopy. Parasite density (asexual parasites per L of blood), was calculated as number of asexual parasites divided by the number of WBCs counted, and then multiplying it by an assumed WBC number (6000 per L). When the number of asexual parasites was<100 per 200 WBCs in follow-up smears, counting was done against at least 500 WBCs. A blood slide sample was considered negative when no asexual parasite seen after examination of 1000 WBCs or 100 fields of thick smear. The presence of gametocytes on the day the patient was enrolled or on the day of follow-up was also recorded.

### Quality assurance of microscopy

Blood smears of enrolled patients, including follow-up smears, were examined by two independent WHO level 1 qualified microscopists. If any discordance was found, a third reading was performed by another senior microscopist. Counting of parasite was also performed by two independent microscopists. If the difference between two readings varied by>25%, counting was again performed by a third microscopist. Each reader was unaware to the result of other reader.

### Data entry and statistical analysis

Data from both clinical and parasitological assessments for each participant were entered and therapeutic efficacy was analysed using WHO standardized Microsoft Excel data collection sheet. Furthermore, survival analysis among total subjects from all study sites was performed using STATA software version 14.

### Molecular analysis

If any patient was found positive for malaria parasite during follow up period, two to three drops of finger prick blood was also collected on 3 MM Whatman filter paper for identification of mixed infection using molecular tools. Genomic DNA was isolated and species-specific nested PCR was carried out using primers targeting the 18s rRNA gene for the identification of *Plasmodium* species [16]. PCR and sequencing of *msp1*, *msp2* and *k13* gene was performed as per previously published protocol [17, 18]. Recrudescence was defined as at least one identical allele for each of the two markers (*msp1* & *msp2*) in the pre-treatment (Day 0) and post-treatment samples (day of parasite observed). Reinfection was diagnosed when all alleles for at least one of the markers differed between the two samples.

### Sequence analysis

Sequencing result were analysed with analysis software v5.2 (Applied Biosystems) and sequence alignment was performed using GeneDoc software [19].

## Results

### Prevalence of malaria and enrolment of study subject

**Fig. 2:**
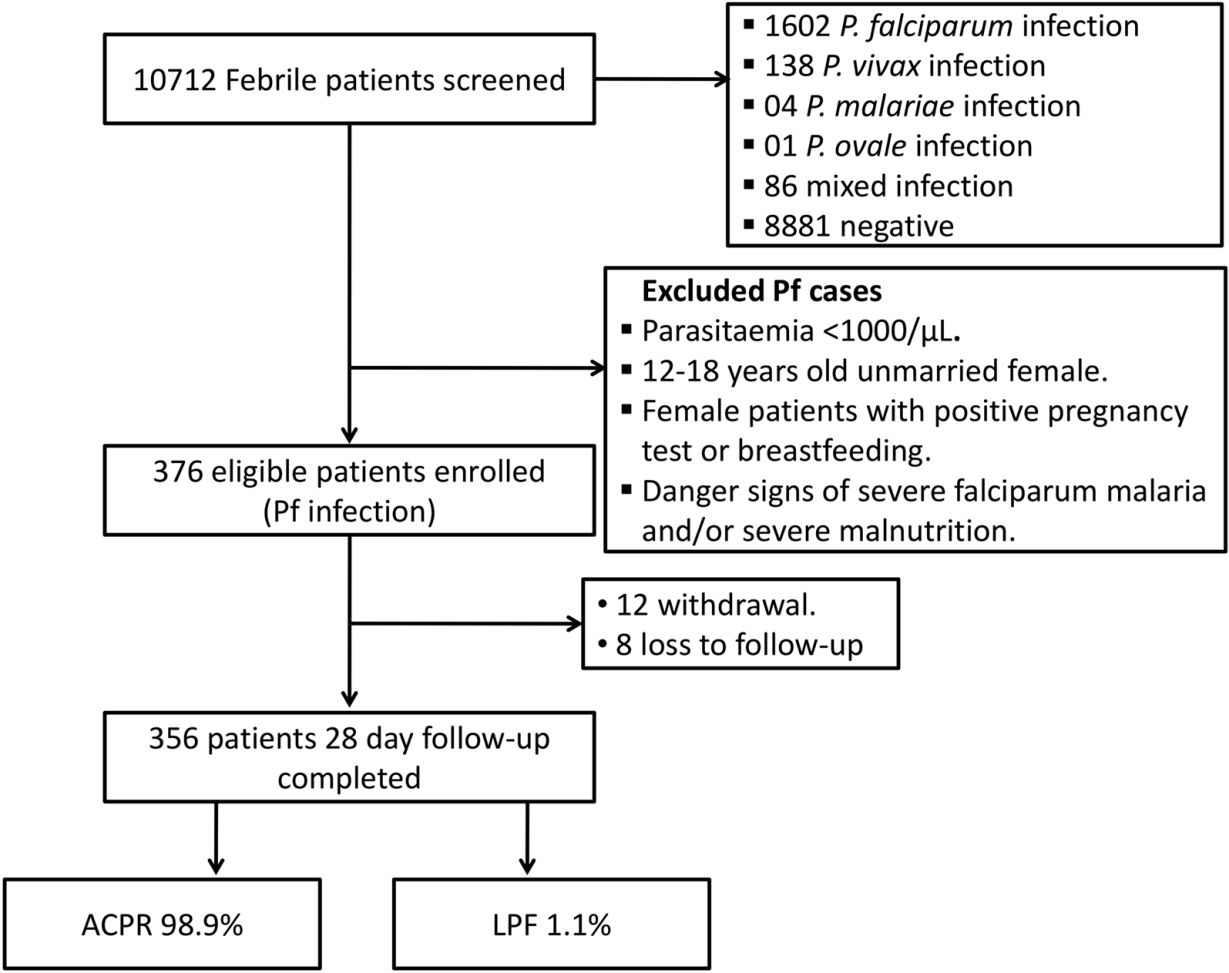
Flowchart showing screening, enrolment and follow-up of malaria patients at all four study sites. ACPR: adequate clinical and parasitological response; LPF: late parasitological failure

Overall, 10,712 patients from 1 to 60years were screened for malaria parasite during the study period. Malaria positivity rate was 17.9% (1915/10712) with 84% (1602/1915) *P. falciparum* monoinfection. A total 12% (224/1915) case of *P. vivax* and 4% (86/1915) mixed infection of *P. falciparum* and *P. vivax* was recorded. Out of 1602 mono *P. falciparum* cases, a total 376 malaria patients who fulfilled the enrolment criteria as well as consented for the study were enrolled. Therapeutic efficacy was determined in 356 (94.7%) patients who had completed their 28days follow-up while 20 patients were either withdrawn from the study or loss to follow up due to various reasons (Fig.2).

### Demographic information of study participant

Background characteristics of the enrolled patients are summarized in Table 1. The median (range) age of the patients was 11years (1year60years). Most of the patients (49%) were 515years old followed by adults (35%) and rest 16% were under 5years and this difference was statistically significant (P<0.001). A similar trend of patients age was observed at each site and no significant difference between the sites was recorded. Overall 62% patients were male and 38% were female which is significant statistically (P<0.0001) and similarly at each site male were significantly more than female. Geometric Mean (95% CI) parasite density at baseline was recorded lowest at Madhya Pradesh (5262.6; 4218.36565.3) followed by Maharashtra (6770.8; 5065.49050.4), Odisha (7380.8; 5480.19940.8) and highest in Chhattisgarh (14,044; 11,617.616,977.2). The difference of mean parasite density between the sites was found significant statistically (P<0.0001).

**Table 1:** Summary statistics of the study participants at four sites

	MP (n=105)	CG (n=117)	MH (n=82)	OD (n=72)	Total (n=376)
Age (years)					
Median	11	10	18	8	11
Mean	15.5254	14.27778	23.37805	11.95833	16.16667
SD	13.27809	10.95415	17.77544	10.49673	13.82476
25th percentile	7	7	8	6	7
75th percentile	19	20	38	14	21
Min.	1	2	1.5	2	1
Max.	59	52	60	50	60
Age group (years)					
Under 5 (%)	12 (11.4)	19 (16.2)	13 (15.9)	17 (23.6)	61 (16.22)
515 (%)	65 (61.9)	58 (49.6)	21 (25.6)	40 (55.6)	184 (48.94)
Adult (%)	28 (26.7)	40 (34.2)	48 (58.5)	15 (20.8)	131 (34.84)
Sex					
Male (%)	49 (46.7)	85 (72.6)	48 (58.5)	50 (69.4)	232 (61.70)
Female (%)	56 (53.3)	32 (27.4)	34 (41.5)	22 (30.6)	144 (38.30)
Weight (kg)					
Median	20	29	40	22	26
Mean	26	31	36	26	29.77287
SD	14.4	15.9	15.7	14.7	15.65803
25th percentile	15	18	22	14.5	16
75th percentile	35	46	47	38	44
Min.	3	6	8	9	3
Max.	65	65	76	69	76
Height (cm)					
Median	125	140	148	120.5	133
Mean	129	136	141	122	132.3378
SD	22.8	28.2	24.5	25.2	26.22478
25th percentile	113	116	124	99	112
75th percentile	148	161	160	146.5	155
Min.	75	48	80	73	48
Max.	170	182	170	168	182
Parasite density/L					
Median	4520	16,320	5920	6958.5	7764.5
Mean	10,912.3	21,995.6	15,757.1	15,901.4	16,373.02
SD	16,912.8	20,083.3	21,535.4	20,995.7	20,151.2
25th percentile	2160	6392	1800	2399.5	2667
75th percentile	11,640	29,980	21,800	20,027.5	22,573.5
Min.	1000	1080	1200	1053	1000
Max.	99,240	98,200	92,280	98,746	99,240
Geometric mean	5262.6	14,044.0	6770.8	7380.8	8051.0
95% CI	(4218.36565.3)	(11,617.616,977.2)	(5065.49050.4)	(5480.19940.8)	(7101.99127.0)

*n* number, *L* micro liter, *MP* Madhya Pradesh, *CG* Chhattisgarh, *MH* Maharashtra, *OD* Odisha

### Efficacy of artemether-lumefantrine

**Fig. 3:**
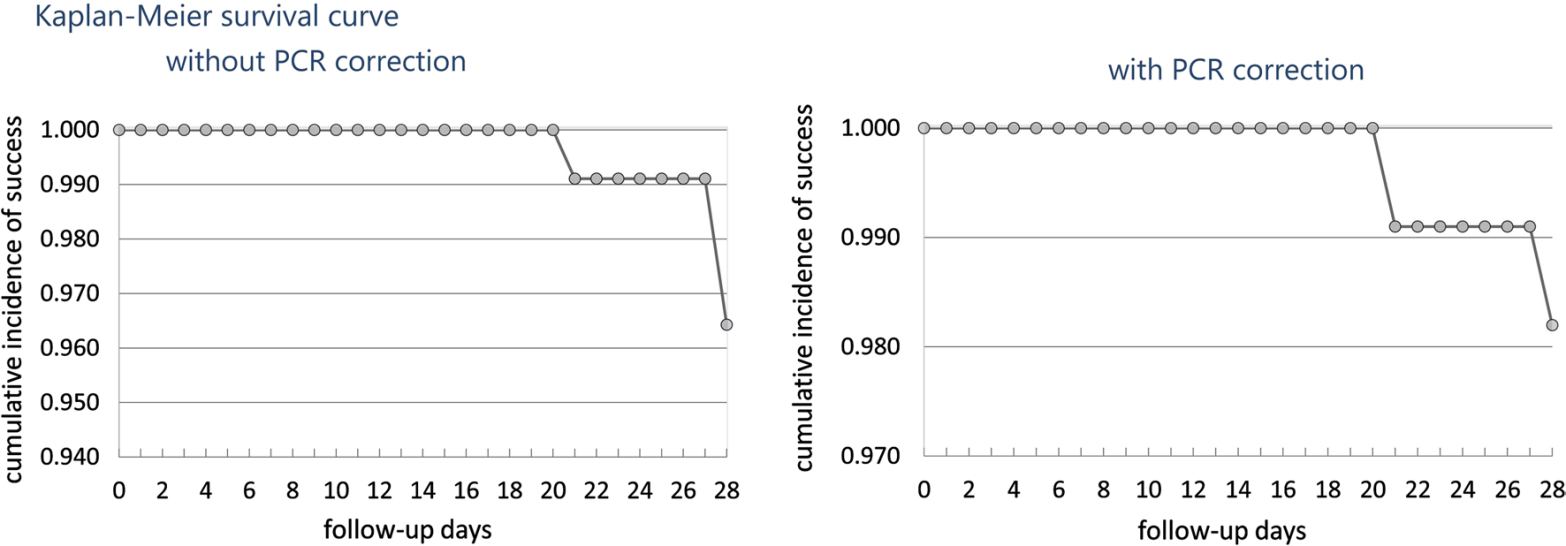
KaplanMeier survival curve showing cumulative incidence of success with (98.2%; 95% CI 9399.5) and without PCR correction (96.4%; 95% CI 90.898.6) at study site Bastar (CG)

**Fig. 4:**
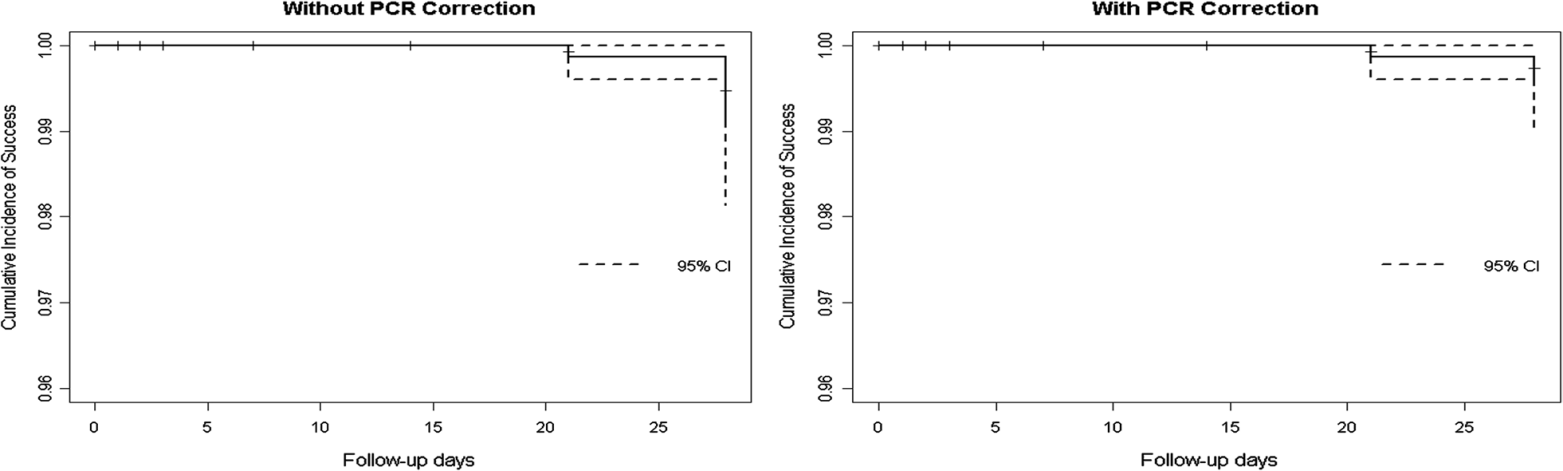
KaplanMeier survival curve showing cumulative incidence of success with (99.4%; 95% CI 97.899.9) and without PCR correction (98.9%; 95% CI 97.199.7) at all study sites

Among the total 376 enrolled cases, 8 patients were loss to follow-up because of remote area and migration of population for their earning and livelihood. Therefore, these patients could not be traced. A total of 12 patients were withdrawn from the study as they were unable to complete the treatment due to persistent vomiting or failure to attend the scheduled visits during the first three days. However, 356 patients were followed up successfully up to 28days. The adequate clinical and parasitological response (ACPR) without PCR correction was 98.9%; 95% CI (97.199.7) with four cases (1.1%; 95% CI 0.32.8) of late parasitological failure (LPF) (Table 2). The ACPR with PCR correction was 99.4%; 95% CI (97.899.9). All four cases of LPF were recorded from study site Chhattisgarh. KaplanMeier survival curve of cumulative incidence of success with and without PCR correction at study site Chhattisgarh is shown in Fig.3. Furthermore, survival analysis curve with and without PCR correction from all the study sites is shown in Fig.4. Neither early treatment failure (ETF) nor late clinical failure (LCF) was observed in this study. Site wise treatment outcomes and parasite clearance time with AL in presented in Table 3. Also, in most of the patients (65%) parasitaemia was cleared within24h. The parasite clearance time was more in the Chhattisgarh and Odisha in comparison to Madhya Pradesh and Maharashtra.

**Table 2:** Site wise summary of patient enrolment and follow up status and treatment result with and without PCR correction

Variables	Study Sites				
	MP	CG	MH	OD	TOTAL
No. of malaria positive cases Screened	251	987	87	506	1831
No. enrolled	105	117	82	72	376
Withdrawal	2	5	0	5	12
Loss to follow-up	0	4	0	4	8
Without PCR correction					
Early treatment failure	0	0	0	0	0
Late clinical failure	0	0	0	0	0
Late parasitological failure	0	4	0	0	4
Adequate clinical and parasitological response (%)	103 (100)	104 (96.3)	82 (100)	63 (100)	352 (98.9)
With PCR correction					
Early treatment Failure	0	0	0	0	0
Late clinical failure	0	0	0	0	0
Late parasitological failure	0	2	0	0	2
Adequate clinical and parasitological response (%)	103 (100)	104(98.1)	82 (100)	63 (100)	352 (99.4)
*Pf* recrudescence	0	2	0	0	0
*Pf* re-infection	0	2	0	0	0
Mixed with Pf recrudescence	0	0	0	0	0
PCR negative (unknown)	0	0	0	0	0

*MP* Madhya Pradesh, *CG* Chhattisgarh, *MH* Maharashtra, *OD* Odisha

**Table 3:** Site wise treatment outcome and parasite clearance time with artemether-lumefantrine

S. no.	Outcome of treatment (28days)	MP	CG	MH	OD	Total
		n (%)	n (%)	n (%)	n (%)	n (%)
1	Primary classification					
	ACPR.	103 (100)	104 (96.3)	82 (100)	63(100)	352 (98.9)
	ETF	0 (0.0)	0 (0.0)	0 (0.0)	0 (0.0)	0 (0.0)
	LCF	0 (0.0)	0 (0.0)	0 (0.0)	0 (0.0)	0 (0.0)
	LPF	0 (0.0)	4 (3.7)	0 (0.0)	0 (0.0)	4 (1.1)
	Loss to follow-up	0 (0.0)	4 (3.4)	0 (0.0)	4 (5.6)	8 (2.1)
	Withdrawal	2 (1.9)	5 (4.3)	0 (0.0)	5 (6.9)	12 (3.2)
	Total	105 (100)	117 (100)	82 (100)	72 (100)	376 (100)
2	Parasite clearance time (hours)					
	24	86 (83.5)	39 (36.1)	79 (96.3)	24(38.1)	228 (64.0)
	>2448	17 (16.5)	68(63.0)	3 (3.7)	37 (58.7)	125 (35.1)
	>4872	0 (0.0)	1 (0.9)	0 (0.0)	2 (3.2)	3 (0.8)
	>72	0 (0.0)	0 (0.0)	0 (0.0)	0 (0.0)	0 (0.0)

*ACPR* adequate clinical and parasitological response, *ETF* early treatment failure, *LCF* late clinical failure, *LPF* late parasitological failure, *MP* Madhya Pradesh, *CG* Chhattisgarh, *MH* Maharashtra, *OD* Odisha

### Molecular analysis of samples with late parasitological failure

Samples from 0day and day of late parasitological failure (LPF) were analysed for *Plasmodium* species by species-specific nested PCR. All the four cases were found positive for *P. falciparum* on both days (0day and day of failure). To understand whether treatment failures were recrudescence or reinfection, DNA samples were sequenced for *P. falciparum* merozoite surface protein 1 and 2. Out of four cases, two contain the same allele for both the genes *msp1* and *msp2* among both time (0day and day of failure), whereas other two patients had re-infection in which *msp1* and *msp2* allele were different at the time of treatment failure.

### Mutations in k13 propeller gene

Total 352 samples were taken for amplification of *k13* gene. Out of these, successful sequencing was done from 308 samples. Alignment of these sequences from reference sequence (PF3D7 1,343,700) showed one Non-synonymous mutation Q613H in one sample from Madhya Pradesh only, while rest of the samples were wild type.

## Discussion

In the present study, efficacy of AL was tested in four states (Madhya Pradesh, Chhattisgarh, Maharashtra and Odisha). These states are major contributor of malaria in India. Among these, Chhattisgarh (18%) and Odisha (11.7%) is the second and third highest contributor of malaria in the country [2]. AL is recommended by National Policy for the treatment of malaria in North Eastern states due to reports of resistance in SP, a partner drug of combination therapy, AS+SP. Although AS+SP is recommended in all other states of the country. Efficacy of AL was assessed in these states, so that AL can be used in case of treatment failure by AS+SP and can be applied by the National Programme in other states as and when required. In the present study, no case of early treatment failure (ETF or ECF) was found, and the ACPR was 98.9%. Previous study carried out three states Madhya Pradesh, Chhattisgarh and Jharkhand in the 2015 also showed that AL is effective in treatment of uncomplicated *P. falciparum* malaria [18]. Moreover, PCR uncorrected efficacy was 100% at all study sites except Chhattisgarh as the enrolled patients were advised to use bed nets (provided by the government) regularly. AL was found equally effective in both the study in 2015 and 2017 among all age groups and areas with high transmission intensity. Cases of *Plasmodium* species mixed infection also have been reported from these study area [20, 21]. Present study results were consistent with other studies from India [22,25], Brazil and Papua New Guinea [26, 27].

Four cases of LPF were observed from Bastar district of Chhattisgarh in the present study. After genotyping, two cases were reinfection and two cases of recrudescence were revealed. Previous study showed 1 case of LCF and 2 cases of LPF from Bastar region while another study from India also reported 1 case of ECF [18, 22]. Parasite resistance to drugs, poor drug absorption and altered pharmacokinetics may result in the treatment failure.

Polymorphism in *k13* propeller gene is associated with artemisinin resistance. In this study, only one non-synonymous mutation (Q613H) was found in one isolate (0.28%) which is not linked to resistance. Previous study by our group from Madhya Pradesh, India showed M579T (1.6%) which is also not linked to artemisinin resistance [18]. Other study showed presence of mutation G533A, S549Y, R561H and A578S in isolate from Tripura, West Bengal, Arunachal Pradesh and Mizoram respectively at low frequency (0.26%) [28]. Among these R561H was reported to associate with delayed parasite clearance [29] and none of these mutations was observed in the present study.

## Conclusion

AL was highly effective for treatment of uncomplicated *P. falciparum* malaria in all age groups and in the areas where more than one *Plasmodium* species are prevalent. Study revealed no functional mutation in *k13* gene. This indicates no immediate threat of artemisinin resistance in study area. However regular monitoring of antimalarial drug efficacy and genotyping is important to tract the emerging resistance and alleles circulating in the malaria endemic areas.

## Data Availability

All data generated or analysed during this study are included in this published article. The datasets used and/or analysed during the current study are available from the corresponding author on reasonable request.
